# Comparative performance of four rapid Ebola antigen-detection lateral flow immunoassays during the 2014-2016 Ebola epidemic in West Africa

**DOI:** 10.1371/journal.pone.0212113

**Published:** 2019-03-07

**Authors:** Betsy Wonderly, Sophie Jones, Michelle L. Gatton, John Barber, Marian Killip, Chris Hudson, Lisa Carter, Tim Brooks, Andrew J. H. Simpson, Amanda Semper, Willy Urassa, Arlene Chua, Mark Perkins, Catharina Boehme

**Affiliations:** 1 Foundation for Innovative New Diagnostics (FIND), Geneva, Switzerland; 2 School of Public Health and Social Work, Queensland University of Technology, Brisbane, Australia; 3 Public Health England, Porton Down, United Kingdom; 4 World Health Organization, Geneva, Switzerland; 5 Médecins Sans Frontières International, Geneva, Switzerland; Etiologic, UNITED STATES

## Abstract

**Background:**

Without an effective vaccine, as was the case early in the 2014–2016 Ebola Outbreak in West Africa, disease control depends entirely on interrupting transmission through early disease detection and prompt patient isolation. Lateral Flow Immunoassays (LFI) are a potential supplement to centralized reference laboratory testing for the early diagnosis of Ebola Virus Disease (EVD).

The goal of this study was to assess the performance of commercially available simple and rapid antigen detection LFIs, submitted for review to the WHO via the Emergency Use Assessment and Listing procedure. The study was performed in an Ebola Treatment Centre laboratory involved in EVD testing in Sierra Leone.

In light of the current Ebola outbreak in May 2018 in the Democratic Republic of Congo, which highlights the lack of clarity in the global health community about appropriate Ebola diagnostics, our findings are increasingly critical.

**Methods:**

A cross-sectional study was conducted to assess comparative performance of four LFIs for detecting EVD. LFIs were assessed against the same 328 plasma samples and 100 whole EDTA blood samples, using the altona RealStar Filovirus Screen real-time RT-PCR as the bench mark assay. The performance of the Public Health England (PHE) in-house Zaire ebolavirus-specific real time RT-PCR Trombley assay was concurrently assessed. Statistical analysis using generalized estimating equations was conducted to compare LFI performance.

**Findings:**

Sensitivity and specificity varied between the LFIs, with specificity found to be significantly higher for whole EDTA blood samples compared to plasma samples in at least 2 LFIs (P≤0.003). Using the altona RT-PCR assay as the bench mark, sensitivities on plasma samples ranged from 79.53% (101/127, 95% CI: 71.46–86.17%) for the DEDIATEST EBOLA (SD Biosensor) to 98.43% (125/127, 95% CI: 94.43–99.81%) for the One step Ebola test (Intec). Specificities ranged from 80.20% (158/197, 95% CI: 74.07–88.60%) for plasma samples using the ReEBOV Antigen test Kit (Corgenix) to 100.00% (98/98, 95% CI: 96.31–100.00%) for whole blood samples using the DEDIATEST EBOLA (SD Biosensor) and SD Ebola Zaire Ag (SD Biosensor). Results also showed the Trombley RT-PCR assay had a lower limit of detection than the altona assay, with some LFIs having higher sensitivity than the altona assay when the Trombley assay was the bench mark.

**Interpretation:**

All of the tested EVD LFIs may be considered suitable for use in an outbreak situation (i.e. rule out testing in communities), although they had variable performance characteristics, with none possessing both high sensitivity and specificity. The non-commercial Trombley Zaire ebolavirus RT-PCR assay warrants further investigation, as it appeared more sensitive than the current gold standard, the altona Filovirus Screen RT-PCR assay.

## Introduction

December 2013 marked the beginning of the most devastating outbreak of Ebola Virus Disease (EVD) in recorded history. Over 20,000 people in Sierra Leone, Liberia and Guinea, were infected, resulting in approximately 11,300 deaths [1]. In the absence of effective drugs, or a vaccine, successful control of the Ebola outbreak depended on prevention of transmission through rapid disease detection, based on symptoms, and timely patient isolation [2]. At Ebola Treatment Centers, patients were typically categorized as suspected or probable cases according to the number and type of symptoms determined during triage [3], and were then isolated in the respective ward until disease was confirmed by laboratory testing. Real time reverse-transcription PCR (RT-PCR) is currently the bench mark method for EVD diagnosis [4]. While sensitive, and capable of detecting viral RNA from both serum and plasma, the large-scale application of this method in an outbreak situation where rapid diagnosis is required for clinical management proved problematic. Specifically, the required manual extraction associated with standard RT-PCR was labour intensive, taking several hours to process [5]. Furthermore, although laboratory processing of individual samples took less than 6 hours, results reporting took up to one week at the height of the outbreak due to the limited laboratory infrastructure, resources and personnel [6]. Ultimately, the use of RT-PCR required significant technical and financial support to maintain, as has been previously described [7].

There was an urgent need for alternative EVD diagnostics, with EVD rapid diagnostic tests (RDTs) being one option to assist with passive and active case finding, contact tracing, triage, cause-of-death investigations, proof of non-contagion, and post-epidemic surveillance [8]. The consensus Target Product Profile for Zaire Ebolavirus detection developed by WHO, FIND, MSF and partners [9] defined the highest priority need and thus this assessment focused on the performance of the RDTs in specific use cases (passive and active detection). EVD RDTs are lateral flow immunoassays (LFI) that exploit antigen-antibody binding technology to enable detection of specific Ebola virus (EBOV) antigens in a clinical sample. Following addition of a positive sample to an LFI, antigen binds to specific dye-labelled antibody; the resultant complexes then migrate along the nitrocellulose test strip, to be bound by a second antigen-specific antibody, thus resulting in appearance of a visible test line [10].

Simple and rapid LFIs offer an attractive alternative approach that is easier and more feasible to implement in decentralized settings, compared to RT-PCR, provided adequate training is provided and the associated biohazard issues can be controlled or minimized [11]. A survey of the technology landscape for Ebola diagnostics was performed in 2014–2015, which identified multiple groups developing, or commercializing EVD LFIs [12]. Using readiness for commercialization criteria, as measured by the World Health Organization (WHO) In Vitro Diagnostic (IVD) Prequalification Team [13], the list of EVD LFIs was narrowed to four. This study was conducted to determine their overall and comparative performance.

While studies had been published previously on the performance of individual EVD LFIs, products were tested using samples from different populations, or collections of archived specimens [14, 15]. Given the broad variability in detectable viral load seen in symptomatic Ebola patients (from 10^2^ to 10^9^ copies/ml) [16] and the variability of RNA viral loads across sample types [17], the performance of a single assay in a single study may largely reflect the type of population enrolled and the sample type included. To overcome this problem, this study set out to compare EVD LFIs against the same sample set, thereby allowing direct comparison of results.

## Methods

### Study design

A blinded, cross-sectional study was conducted to determine comparative performance of four EVD LFI antigen detection tests against anonymised archived residual diagnostic plasma and fresh venous EDTA blood specimens. The study was conducted at the Public Health England (PHE) laboratory at the Makeni (Mateneh) Ebola Treatment Centre (ETC) in Makeni, Bombali district, Sierra Leone [18].

Biosafety precautions according to PHE Laboratory Standard Operating Procedures (SOPs) were followed throughout the assessment. Ethical approval was obtained from the WHO Ethical Review Board and the Sierra Leone Ethics and Scientific Review Committee (Study ID: PQDx_189_V4.0) for the comparative assessment WHO protocol. There was no study-related case follow-up and results were not used for patient care.

### Samples

Sample panels comprised 100 prospectively collected venous EDTA blood samples sourced directly from the Makeni ETC as patients were admitted from March 27, 2015 –May 9, 2015, and 344 retrospectively tested plasma samples, which had been stored at -80°C, at either the Nigeria Mobile Laboratory in Kambia, the European Mobile Laboratory in Hastings, or the PHE ETC laboratories in Kerrytown, Port Loko and Makeni. The 344 archived samples were selected across the entire range of viremia (using PCR Ct value as a surrogate measure of viral load), thus representing populations seen in 1) passive case-finding (i.e. EVD identified in symptomatic patients presenting at an ETC; high-viral load) and 2) active case-finding (i.e. EVD identified in individuals sought by healthcare workers among case contacts and other at-risk individuals in the field; low viral load).

Due to the large sample volume required to perform four LFIs and two RT-PCR assays, the majority of archived specimens were pooled according to origin, Ct value, and haemolysis severity. Once samples were thawed for pooling, they were used immediately for all experiments, then disposed of. All sample pooling and aliquoting, along with performance of the LFIs, was conducted inside a negative air pressure, HEPA exhaust filtered, flexible film isolator (VersarPPS, Milton Keynes, UK), in accordance with PHE SOPs.

### LFI tests and evaluation method

In September 2014, WHO launched the Emergency Use Assessment and Listing (EUAL) procedure, to respond to the urgent need for diagnostic tests during the unprecedented EVD outbreak in West Africa [19]. Manufacturers were invited to submit manufacturing quality management documentation and a product dossier for promising technologies to detect EBOV antigens. Quality management documentation and product dossier review was coordinated by WHO. Four LFIs, which met the initial review QMS criteria and aligned with the WHO Target Product Profile [9] were approved for limited laboratory evaluation of performance. The products are listed in Table 1.

**Table 1:** LFI tests approved for limited evaluation of performance by WHO.

	LFI Test Name	Manufacturer	IFU Version	Antigens Targeted
1	One step Ebola test	Intec	CAT. NO. ITP08001, REV. 150201	Not specified
2	DEDIATEST EBOLA	Senova	2.13.1.109	VP40
3	ReEBOV Antigen test Kit	Corgenix	13981 01	VP40
4	SD Ebola Zaire Ag	SD Biosensor	Feb 2015 Version	GP, NP, VP40

Products included in the assessment were from a single manufacturing lot and were shipped from manufacturers in climate-controlled conditions with temperature loggers. No shipments were exposed to temperatures outside the manufacturer specified requirements during shipping, or during storage in Sierra Leone.

LFI evaluations were conducted according to the manufacturer instructions, with the following deviations:

1. One step Ebola test (Intec). Precision pipette was used to deliver 100 μL of sample, instead of the provided transfer device.
2. DEDIATEST EBOLA (Senova). i) Venous EDTA-treated whole blood was tested (which was not listed as a sample type option in the Instructions For Use (IFU)) following manufacturer approval. ii) Haemolyzed samples were included in the study. iii) Precision pipette was used to deliver 100 μL of sample to extraction tube, instead of the provided transfer pipette. The transfer pipette was however used to transfer sample/buffer mix from extraction tube to cassette according to the IFU.
3. ReEBOV Antigen test Kit (Corgenix). Haemolyzed samples were included.
4. SD Ebola Zaire Ag (SDBiosensor). Haemolyzed samples were included.

Two manufacturers, SD Biosensor and Corgenix, provided positive and negative controls, which were tested on all LFIs at the following time points: two weeks after study launch, four weeks after study launch, and three weeks prior to study conclusion. The study duration was approximately three months. The objective was to provide internal validation that no apparent LFI degradation had occurred. No abnormal results were obtained during this testing.

Two technicians independently interpreted each LFI result. A test was declared positive when visible control and test bands were observed. A negative result was identified as a visible control band, but no visible test band. Absence of a control line, irrespective of the presence of a test band, was recorded as an invalid test. The technicians were blinded to each other’s results. Test interpretation discrepancies (i.e. valid versus invalid and positive versus negative) were resolved by a third reader. When an invalid test result occurred, the test was repeated provided there was sufficient sample remaining.

All data were recorded on paper data collection forms, and then double entered into an Excel spread sheet by two technicians.

### RNA extraction for RT-PCR

**Fig 1:**
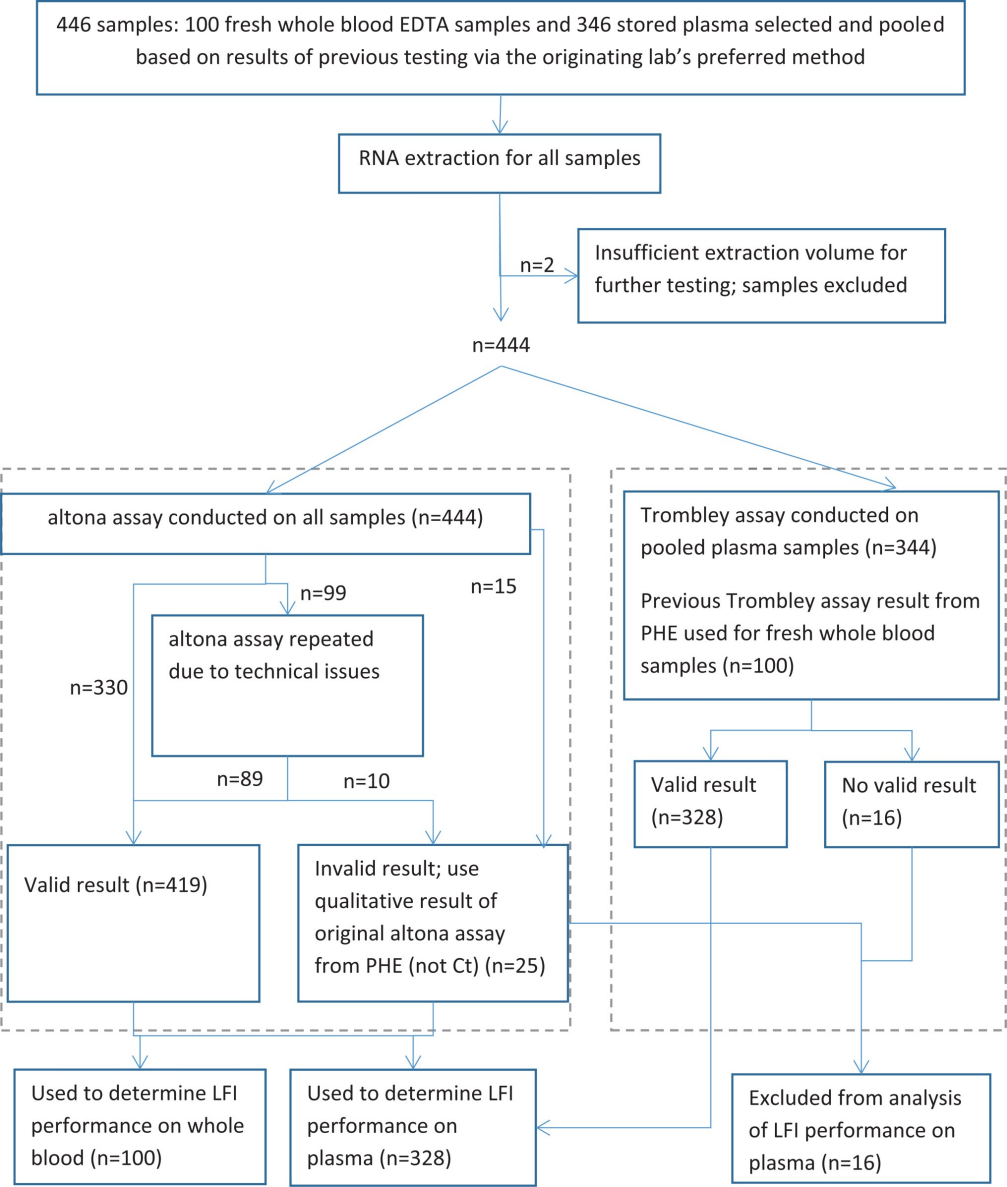
Flow chart of PCR analysis of samples. altona assay = RealStar Filovirus Screen RT-PCR kit 1.0; Trombley assay = PHE Zaire-specific RT-PCR Trombley assay.

The study profile in Fig 1 depicts the sample extraction and RT-PCR amplification strategy for this study.

RNA was manually extracted from 140 μL plasma (either pooled archived plasma or freshly prepared from whole EDTA blood samples) using the QIAamp Viral RNA kit (Qiagen Inc., USA) according to the manufacturer instructions. For 56 (12.6%) of the 444 samples, 8 (8%) originating from whole EDTA blood and 48 (14%) from stored plasma, there was insufficient plasma for the full 140 μL extraction so a smaller volume, typically 80 μL, was used. Each batch of extractions included a negative extraction control comprising 140 μL PCR grade water. 9 μL of template internal control from the RealStar Filovirus Screen RT-PCR kit 1·0 was included in each reaction destined for EBOV RT-PCR using the altona assay; 24 μL MS2 phage was included as exogenous internal control in each extraction destined for EBOV RT-PCR using the Trombley assay. All extracts were eluted in 60 μL AVE buffer.

### Real time RT-PCR

10 μL of each sample extract and negative extraction control was subjected to real-time RT-PCR for detection of EBOV using the altona RealStar Filovirus Screen RT-PCR kit 1.0 (target: L gene and hereafter referred to as the altona assay) according to manufacturer instructions [20], on a Lightcycler 96 Real-Time PCR system (Roche Diagnostics, Risch-Rotkreuz, Switzerland).

The PHE Zaire ebolavirus-specific RT-PCR Trombley in-house assay [21] (target: NP gene and hereafter referred to as the Trombley assay) was carried out as described previously [13]. This assay was performed on RNA extracts from the 100 fresh whole blood samples by staff working in the PHE Makeni laboratory [21]. Due to limited sample volumes, PHE results were used for this study, rather than repeating the assay. For the 344 retrospective plasma samples, the Trombley assay was re-run by study staff.

For both the altona and Trombley RT-PCR assays, samples with Ct ≥ 40 and a positive internal control were interpreted as EBOV negative; if the internal control failed, the result was interpreted as a sample failure, and the test was repeated. Samples with Ct > 0 and < 40 with or without a positive internal control were interpreted as EBOV positive.

### Statistical analysis

Data were analysed in IBM SPSS Statistics (Version 22.0, IBM Corporation). Pearson’s correlation was used to determine the association between Ct values from the altona and Trombley RT-PCR assays, and a paired t-test was used to test whether the Ct values were significantly different. The difference in Ct values between matched samples were compared between various sample groupings using t-tests. Median Ct values were compared between haemolyzed and non-haemolyzed samples using the Median Test.

The relative performance of LFIs, specifically the sensitivity and specificity on plasma samples and specificity on whole blood samples, were compared using generalized estimating equations (GEE) with a log link function, using sample as the repeat unit. In these models sensitivity (or specificity) was the outcome and LFI product was the factor of interest, with potential confounders such as sample type and Ct value, controlled for.

## Results

All 100 prospectively collected whole EDTA blood samples tested negative for EBOV RNA by both RT-PCR assays. Therefore these samples were only used to determine LFI specificity. A flow chart of the PCR analysis of samples is depicted in Fig 1.

Approximately half (50.7%) the samples showed some degree of haemolysis, with 53.0%, 33.2% and 13.7% of these classified as mild, moderate and heavy haemolysis, respectively.

### PCR results

A total of 135 (30.4%) samples were classified as positive and 309 (69.6%) as negative using the altona assay. Ct values were available for 126/135 positive samples with values ranging from 15.2 to 33.7 with a median of 22.4. Using the Trombley assay 156 (47.6%) samples were positive, with the Ct values ranging from 14.0 to 37.7 with a median of 22.3. Overall, haemolysed samples had significantly lower median Ct values on plasma samples for both RT-PCR assays; 21.8 (IQR 6.2) and 24.9 (IQR 6.1) by altona assay and 20.9 (IQR 8.1) and 27.5 (IQR 11.0) by Trombley assay, for haemolysed and non-haemolysed samples respectively (P<0.003).

A total of 328 plasma samples had results from both RT-PCR assays. All samples that were positive using the altona assay were also positive using the Trombley assay, however the reverse was not true (Table 2). The sensitivity and specificity of the altona assay compared to the Trombley assay were 82.69% (129/156; 95% CI: 75.83–88.27%) and 100.00% (172/172; 95% CI: 97.88–100.00%), respectively. Ct values obtained from the altona and Trombley assays were strongly correlated (n = 126, r = 0.958, P<0.001). On average, Ct values obtained using the Trombley assay were 1.5 units (95% CI: 1.3–1.8) lower than the altona Ct value on the same sample (P<0.001). This difference was not influenced by Ct value (P = 0.35) or sample haemolysis (P = 0.65).

**Table 2:** Comparison of altona and Trombley RT- PCR results.

		Trombley PCR		
		PCR negative	PCR positive	Total
altona PCR	PCR negative	172	27	199
	PCR positive	0	129	129
	Total	172	156	328

Twenty-seven samples were negative using the altona assay but positive using the Trombley assay (Table 2). These 27 samples had a significantly higher mean Trombley Ct value (mean = 34.2, sd = 1.9) than the 129 samples that were positive using the altona assay (mean = 21.9, sd = 4.4) (P<0.001).

### Performance of LFIs

Overall fewer than 3% (51/1766) of LFIs tested required a third read to resolve discrepant results. SD Ebola Zaire Ag (SD Biosensor) and DEDIATEST EBOLA (Senova) had the lowest and highest discrepancy rates with 1.1% (5/441) and 5.5% (24/440) of tests, respectively. Where a third read was required, the discrepancy involved one technician reporting a control or test band as negative and the other technician recording a faint positive band.

The SD Ebola Zaire Ag (SD Biosensor) had the highest invalid rate of 2.9% (13/454, total number of tests includes repeat testing of invalid tests). Invalid rates for the remaining three LFIs were similar; 0.9% (4/448) for ReEBOV Antigen test Kit (Corgenix), 0.9% (4/445) for One step Ebola test (Intec) and 1.3% (6/446) for DEDIATEST EBOLA (Senova). Failure to flow was the cause of all invalid tests for SD Ebola Zaire Ag and ReEBOV Antigen test Kit (Corgenix). For One step Ebola test (Intec), failure to flow was responsible in 3/4 invalid tests, while there was no visible control in the remaining invalid test. For DEDIATEST EBOLA (Senova), failure to flow was responsible for 2/6 invalid tests. In the four other tests where whole EDTA blood was used, the background was too dark, and obscured the control band.

### LFI performance compared to altona RT-PCR

Sensitivity and specificity varied between the LFIs, and also by sample type (Table 3). Statistical analysis revealed a significantly higher specificity in whole EDTA blood samples compared to plasma samples for ReEBOV Antigen test Kit (Corgenix) (p = 0.001) and One step Ebola test (Intec) (p = 0.003). This analysis could not be performed for SD Ebola Zaire Ag (SD Biosensor) or DEDIATEST EBOLA (Senova) products, since there were no false positive results against the whole EDTA blood samples.

**Table 3:** Performance of LFIs compared to altona RT-PCR*.

	Sensitivity on plasma samples (95% CI)†	Specificity (95% CI)	
		Whole EDTA blood	Plasma†
SD Ebola Zaire Ag	109/129	97/97	196/198
	84.50 (77.08–90.27)^a^	100.00 (96.27–100.0)	98.99 (96.40–99.88)^c^
ReEBOV Antigen test Kit	123/129	98/100	159/198
	93.18 (87.45–96.84)^b^	98.00 (92.96–99.76)	80.30 (74.07–88.60)^d^
One step Ebola test	125/127	95/100	158/197
	98.43 (94.43–99.81)^b^	95.00 (88.72–98.36)	80.20 (73.95–85.53)^d^
DEDIATEST EBOLA	101/127	98/98	167/198
	79.53 (71.46–86.17)^a^	100.00 (96.31–100.0)	84.34 (78.52–89.11)^d^

*LFI products with different superscripts have a statistically significant difference in sensitivity or specificity (P<0.05).

†Only includes samples that have results for both altona and Trombley PCR assays.

The LFI results against plasma samples indicated that the ReEBOV Antigen test Kit (Corgenix) and One step Ebola test (Intec) products had significantly higher sensitivity than SD Ebola Zaire Ag (SD Biosensor) and DEDIATEST EBOLA (SD Biosensor) (p<0.001), and that SD Ebola Zaire Ag (SD Biosensor) had significantly higher specificity than the other 3 LFIs (p<0.001) (Table 3).

**Fig 2:**
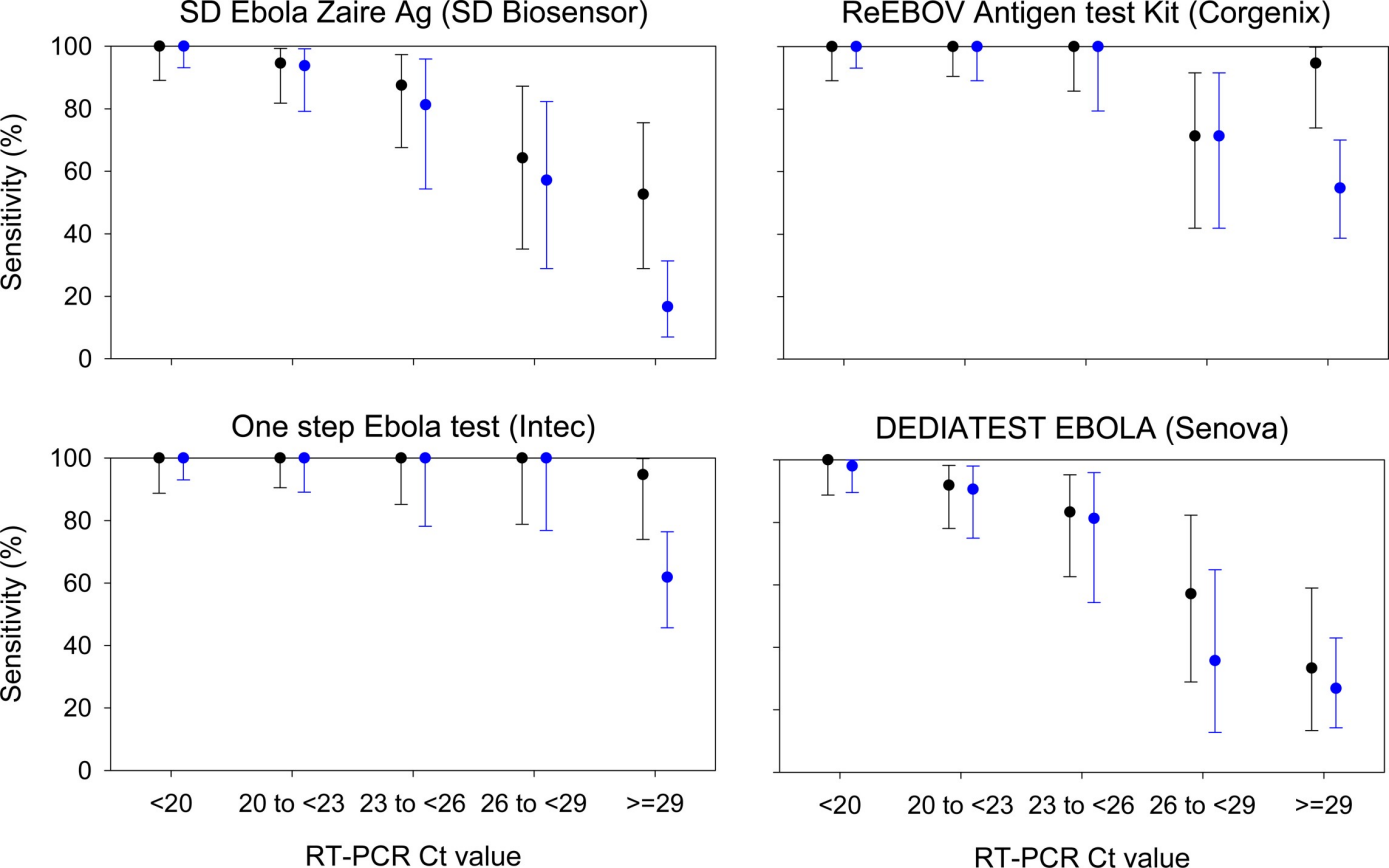
Sensitivity of LFIs for samples grouped according to altona Ct value (black) and Trombley Ct value (blue). Bars represent 95% confidence intervals for sensitivity. For the altona assay there were 32, 37, 24, 14 and 19 samples in each RT-PCR Ct group from <20 to ≥29. The corresponding sample sizes for the Trombley assay were 52, 32, 16, 14 and 42.

LFI sensitivity was influenced by sample viral load, as represented by Ct value (Fig 2). The relationship between sensitivity and Ct value could not be formally tested due to all LFIs having 100% sensitivity at low Ct values. However there was a clear dose response, which differs between LFI products (Fig 2).

### LFI performance compared to Trombley PCR

LFI sensitivity compared to the Trombley assay fell into two distinct groups; ReEBOV Antigen test Kit (Corgenix) and One step Ebola test (Intec) had significantly higher sensitivity than SD Ebola Zaire Ag (SD Biosensor) and DEDIATEST EBOLA (Senova) (p<0.001) (Table 4).

**Table 4:** Performance of LFIs on plasma samples using Trombley assay as bench mark assay*.

	Sensitivity (95% CI)	Specificity (95% CI)
SD Ebola Zaire Ag	110/156	170/171
	70.51 (62.69–77.53)^a^	99.42 (96.78–99.99)^c^
ReEBOV Antigen test Kit	133/156	142/171
	85.26 (78.70–90.42)^b,c^	83.04 (76.56–88.34)^d^
One step Ebola test	138/154	144/170
	89.61 (83.68–93.94)^b^	84.71 (78.40–89.76)^d^
DEDIATEST EBOLA	108/154	147/171
	70.13 (62.24–77.23)^a^	85.96 (79.84–90.80)^d^
altona PCR	129/156	172/172
	82.69 (75.83–88.27)^c^	100.00 (97.88–100.00)

* Only samples with RT-PCR results for both altona and Trombley assays are included. LFI products with different superscripts for sensitivity or specificity have a statistically significant difference in performance (p<0.05)

SD Ebola Zaire Ag (SD Biosensor) had significantly higher specificity than any of the other 3 LFIs (p<0.003), which were not significantly different from each other (p>0.4) (Table 4). These results mirror those obtained using altona assay as the bench mark assay (Table 3). Incorporating results of the altona assay (relative to Trombley assay) into the statistical model indicated the sensitivity of the One step Ebola test (Intec) was significantly higher than the altona assay (p = 0.006), while the altona assay sensitivity was comparable to ReEBOV Antigen test Kit (Corgenix) (Table 4).

There was also a clear relationship between LFI sensitivity and Trombley assay Ct value; which was similar to that seen using altona PCR as the bench mark (Fig 2).

## Discussion

The development of diagnostic tests which can be easily and rapidly deployed in emergency situations is a high priority for future EVD case management and outbreak containment. Although EVD has existed for many decades, the unprecedented size of the recent outbreak in West Africa highlighted the potentially explosive nature of the disease and devastating personal and social consequences associated with quarantining large numbers of suspected cases until diagnostic results can be obtained. In response to this need, a number of commercial LFIs for the detection of Ebola virus antigen were developed. This study provided a unique opportunity to directly compare the performance of four these LFIs using a single sample set in preparation for future EVD outbreaks.

The LFIs examined demonstrated variable performance; no single one had both high sensitivity and high specificity. Three of the four LFIs showed reduced specificity when tested using plasma samples compared to whole EDTA blood, indicating that separate specificity values should be reported for each sample type. It should be noted that only SD Ebola Zaire Ag (SD Biosensor) and ReEBOV Antigen test kit (Corgenix) state whole blood specifically as the recommended sample type. The DEDIATEST EBOLA (Senova) listed serum and throat swab as the validated sample types. One step Ebola test (Intec) listed serum, plasma, and whole blood as possible sample types. It is interesting that the specificity on the whole blood sample type was higher than for plasma samples.

Combining the results for sensitivity and specificity on plasma samples, the LFIs can be broadly categorised into three groups:

- SD Ebola Zaire Ag (SD Biosensor) with moderate sensitivity and the highest specificity
- ReEBOV Antigen test Kit (Corgenix) and One step Ebola test (Intec) which had the highest sensitivity but lowest specificity, and
- DEDIATEST EBOLA (Senova) with the lowest sensitivity and moderate specificity

In deciding which LFIs are adequate for field deployment in remote locations it is important to consider biosafety, storage requirements and ease of use of the individual LFI tests, in addition to performance. For example, the ReEBOV Antigen test Kit (Corgenix) required storage at 2–8°C which would be challenging for most field situations. In addition, operator biosafety concerns were noted for three of the LFIs: i) DEDIATEST EBOLA (Senova) as the sample is added to the open reaction tube for initial incubation and the reaction tube holder was not stable, ii) ReEBOV Antigen test Kit (Corgenix) as after adding the sample to the test strip, the strip must be picked up by the operator and placed in the reaction tube, and iii) SD Ebola Zaire Ag (SD Biosensor) which frequently exhibited excess pooling on the sample reception pad. Modifying the LFI test procedure to avoid these concerns would increase the utility of these tests for emergency field deployment.

An interesting and important finding of this study is the systematic difference in the results of the Trombley and altona RT-PCR assays used as benchmarks. The difference between the Ct values of the assays (On average, Ct values obtained using the Trombley assay were 1.5 units (95% CI: 1.3–1.8) lower than the altona Ct value on the same sample (P<0.001)) was consistent across the range of sample viral loads and was not affected by sample haemolysis. All positive samples showed a lower Ct value on the Trombley assay compared to the altona assay and the subset of samples that were negative according to the altona assay but positive according to the Trombley assay had a significantly higher mean Trombley Ct value compared to those that were positive on the altona assay. Hence it appears that the difference in positivity between the two RT-PCR methods is attributable to samples with low viral load, and that the Trombley assay has a lower limit of detection. It is important to note that the choice of equipment and processes used in the PHE-led laboratories was made to meet operational needs for rapid clinical deployment in a humanitarian outbreak scenario. Thus, despite the fact that the Lightcycler 96 was not recommended as a validated platform for the altona assay, processing and platform choices that might be optimal in controlled laboratory conditions can be impractical under field conditions during an outbreak. The manufacturer was consulted prior to used, however, these results should be further investigated, and perhaps the analytical detection limit of each assay (Trombley, altona, and the LFIs) determined, as it may have significance for the clinical diagnosis of samples. Currently the Trombley assay is an in-house assay so further investigation is also required to explore whether the manufacture of this assay can be scaled up in a commercial setting.

Another key challenge in conducting this study was gaining access to EVD samples. Ideally this study of LFIs would have been conducted solely using fresh patient samples however the study was conducted towards the end of the epidemic when the number of EBV positive cases was very limited. In addition patient samples were very difficult to gather for the evaluation, due to challenges related to sample ownership, and the economics of this novel disease outbreak setting: minimal specimen supply and high sample demand from the research community. As a result archived samples were included in the study to achieve an adequate sample size for statistical analysis. It is recommended that clear guidelines pertaining to sample ownership be developed as soon as practical, prior to future outbreaks. In addition, early organization of a specimen bank and a published list of agreed research priorities may ensure that limited resources are distributed to achieve maximum public health impact.

This study had several limitations, which should be considered when interpreting the results. As mentioned previously, it was difficult to access fresh whole EDTA blood samples. As a result, the samples used had the following limitations: 1) we couldn’t carry out the study in the hot zone on freshly drawn venous blood 2) The fresh blood we could access was in the PHE lab and was EDTA treated and 3) When we were able to access EDTA-blood, it all came from EVD negative cases and was of no use for the sensitivity calculations. The samples obtained were often of low volume due to extreme dehydration of patients reporting for EVD testing. As a result, this assessment included haemolyzed samples, samples with varying plasma volume, and no EVD positive whole EDTA blood samples. Interestingly, although the LFIs were not recommended for use on haemolyzed samples, they performed well on these samples. This may be due to the fact that haemolyzed samples typically were derived from patients with lower Ct values and thus higher EBOV antigen levels.

Another limitation was small but necessary deviations from the manufacture-specified instructions and equipment. The assessment was completed by operators in full PPE, with their arms inside a small FFI, donning a double layer of gloves, in an environment with high temperatures. A limited number of supplies could be packed into the FFI and the operators could only remain working in the FFI for a limited period of time. As a result, some modifications had to be made to the LFI IFU (e.g. using a common precision pipette vs the manufacturer-provided transfer pipette, which takes up more space). Although we do not believe any of these modifications impacted the results of the study, they should be noted.

In conclusion, the LFIs for EVD have variable performance, with no single test showing both high sensitivity and high specificity. Although their performance, pertaining to specificity in particular, is superior to the other LFIs, the environmental shipping requirements for the ReEBOV Antigen test Kit (Corgenix) and the biohazard risk of the SD Ebola Zaire Ag (SD Biosensor) should be considered prior to use in the field.

The results of this study were referenced in the development of the WHO emergency guidance—Selection and use of Ebola in vitro diagnostic (IVD) assays—during the latter half of the outbreak [22]. To the best of the authors’ knowledge, a consensus still needs to be reached regarding how such LFIs would be used in an EVD outbreak with varying levels of disease prevalence. Would they be used for rapid triage at ports of entry and ETCs to support case segregation? Would they be used for case finding? Would a single test be used or two different tests? A few guidance documents [23, 24] released by the WHO could be helpful tools if used in combination with performance data for each assay to develop such guidance in advance of future outbreaks.
